# An Open-Hardware Insemination Device for Small-Bodied Live-Bearing Fishes to Support Development and Use of Germplasm Repositories

**DOI:** 10.3390/ani12080961

**Published:** 2022-04-08

**Authors:** Elise R. Harmon, Yue Liu, Hamed Shamkhalichenar, Valentino Browning, Markita Savage, Terrence R. Tiersch, William Todd Monroe

**Affiliations:** 1 Department of Biological and Agricultural Engineering, Louisiana State University, Baton Rouge, LA 70803, USA; eliseharmon1@gmail.com (E.R.H.); yliu@agcenter.lsu.edu (Y.L.); vdudz1234@gmail.com (V.B.); 2 Aquatic Germplasm and Genetic Resources Center, School of Renewable Natural Resources, Louisiana State University Agricultural Center, Baton Rouge, LA 70820, USA; hamed.shamkhali@gmail.com (H.S.); ttiersch@agcenter.lsu.edu (T.R.T.); 3 School of Electrical Engineering and Computer Science, Louisiana State University, Baton Rouge, LA 70803, USA; 4 The *Xiphophorus* Genetic Stock Center, Texas State University, San Marcos, TX 78666, USA; markita@txstate.edu

**Keywords:** live-bearing fish, viviparous, artificial insemination, sperm, cryopreservation, standardization, open hardware

## Abstract

**Simple Summary:**

Small-bodied live-bearing fishes attract broad attention because of their importance in biomedical research and critical conservation status in natural habitats. Artificial insemination is an essential approach used to establish hybrid lines and for the operation of sperm repositories. The existing mouth-pipetting technique for artificial insemination of live-bearing fishes has not been substantially upgraded since the first implementation in the 1950s. This work developed a low-cost standardized artificial inseminator device (SAID) as open hardware to address issues routinely encountered in insemination by mouth-pipetting, including lack of reproducibility among different users, difficulty in training, and large, unreportable variation in sample volume and pressure during insemination. Community-level enhancements of the SAID prototype could enable standardized insemination with minimal training and facilitate the participation of research communities in the use of cryopreserved genetic resources.

**Abstract:**

Small-bodied live-bearing fishes attract broad attention because of their importance in biomedical research and critical conservation status in natural habitats. Artificial insemination is an essential process to establish hybrid lines and for the operation of sperm repositories. The existing mouth-pipetting technique for artificial insemination of live-bearing fishes has not been substantially upgraded since the first implementation in the 1950s. The goal of this work was to develop a standardized artificial inseminator device (SAID) to address issues routinely encountered in insemination by mouth-pipetting, including lack of reproducibility among different users, difficulty in training, and large unreportable variation in sample volume and pressure during insemination. Prototypes of the SAID were designed as relatively inexpensive (<USD 80) open hardware based on commercially available and 3-D printed components to enable broad community access. A linear actuator was used to accurately control the position of a piston for fluid transfer with a standard deviation of <0.1 mm over a 4 mm range of travel. The volume of sample transfer was precisely controlled with a linear relationship (*r*^2^ > 0.99) between the piston position and volume. Pressure generation from eight mouth-pipetting operators and SAID prototypes were assessed by pressure sensors. The pressure control by SAID was superior to that produced by mouth-pipetting, yielding lower pressures (31–483 Pa) and smaller variations (standard deviation <11 Pa). These pressures were sufficient to deliver 1–5 μL of fluid into female reproductive tracts yet low enough to avoid physical injury to fish. Community-level enhancements of the SAID prototype could enable standardized insemination with minimal training and facilitate the participation of research communities in the use of cryopreserved genetic resources.

## 1. Introduction

Small-bodied (<10 cm in length) live-bearing (viviparous) fishes have been widely recognized as research models for decades [1,2]. For example, *Xiphophorus* species have been used extensively to study spontaneous and induced tumorigenesis [3,4,5]. The biomedical importance of these species led to the establishment of the *Xiphophorus* Genetic Stock Center (XGSC, xiphophorus.org), which maintains and distributes more than 60 pedigreed lines derived from 24 of the 26 species within the genus. Additionally, *Xiphophorus* are commonly used for studies in behavioral ecology [6,7], virology [8], evolutionary biology [9,10], and conservation [2,11]. However, small live-bearing fishes are one of the most at-risk freshwater taxa in the world [12,13]. The family Poeciliidae, the largest live-bearing freshwater fish group, includes 81 species assessed by the International Union for Conservation of Nature (IUCN, iucnredlist.org) as being threatened, vulnerable, endangered, or extinct. In the family Goodeidae, 37 of the 40 species are considered to be of concern [14]. This combination of conservation concern and long-standing value to research has driven the emergence of sperm cryopreservation and the development of germplasm repositories for these fishes [12,15]. 

Artificial insemination is an essential step to evaluate and use cryopreserved sperm in live-bearers [12,16] and is a critical procedure for the creation of the hybrid lines used for genetic research [17]. Existing approaches by use of mouth-pipetting for artificial insemination of live-bearing fishes are essentially unchanged since the 1950s [18]. Mouth-pipetting has been used to produce live young with cryopreserved sperm for *Xiphophorus helleri* [15], critically endangered *X. couchianus* [19], *X. maculatus* [20], *Poecilia reticulata* [21], *P. latipinna* [21], and the endangered *Xenotoca eiseni* [22]. Typically, at the XGSC, 50–60% of females that are inseminated with fresh sperm (10–20% of females that are inseminated with cryopreserved sperm) produce live young with female mortality of 10–20% within the first month of insemination (unpublished data from XGSC spanning 10 years and 190 inseminated females). It is important to note that this represents an upper level of success because inseminations are performed at the XGSC by well-trained staff with years of experience. Most laboratories among the research community do not have well-trained technicians, posing substantial challenges in the use of cryopreserved resources. Improvement of standardized insemination hardware could increase fertilization, facilitate resource utilization, increase reproducibility, and reduce fish mortality and training costs.

**Figure 1:**
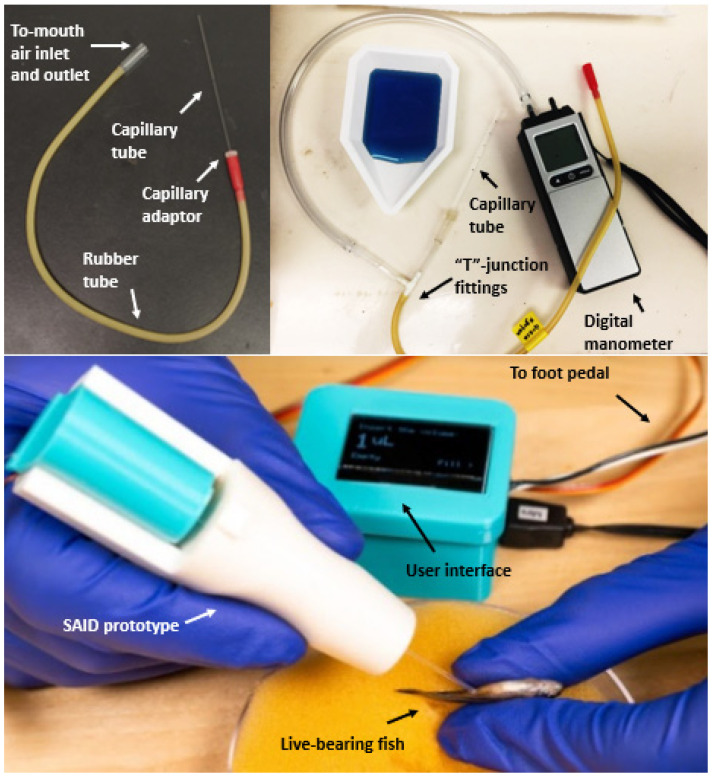
Comparison of the existing mouth-pipetting tool [31] and the standardized artificial insemination device (SAID) for use with small-bodied live-bearing fishes. A standard mouth-pipetting tool (**upper left**) consists of a capillary that fits into a rubber tube with adaptor fittings. Pressures generated during mouth-pipetting were characterized by use of a digital manometer (**upper right**) with a “T”-junction fitting. A prototype of the SAID (**bottom**) is shown during injection of samples into the reproductive tract of a female live-bearing fish.

In the traditional mouth-pipetting method (Figure 1), fluid flow is controlled by exhalation pressure with the mouth at one end of a rubber tube and a capillary tube at the other end to deliver sperm into the female genital opening. Major technical problems of this method are the lack of standardized control of volume, pressure, and flow rate. The insemination volume of sperm samples for small-bodied live-bearing fishes is <5 µL [22], and thus, small variations in breathing pressure can lead to relatively large variations in fluid delivery. For example, during insemination of 2 µL of suspension at a concentration of 2 × 10^6^ sperm/mL [21], the difference of a single microliter can cause a variation of ~10^6^ sperm being injected into the female, which can produce variations in fertilization rate [23]. Therefore, it is almost impossible to compare research results across different laboratories or operators to reproduce the outcomes of protocols. In addition, unstandardized pressure can result in variations in depth of sperm delivery into the female reproductive tract that can affect fertilization rate, and excessive pressure applied to the reproductive tract can cause injury or mortality to female fish.

In recent years, open hardware has become an emerging strategy to share technologies that can support scientific research [24,25] and facilitate reproducibility and participation among community members [26,27]. With this strategy, hardware is distributed through the open sharing of design files and low-cost standardized fabrication [28,29,30]. The goal of this work was to develop a low-cost standardized artificial inseminator device (SAID) as open hardware for small-bodied live-bearing fish. The objectives were to: (1) design and fabricate inseminator prototypes; (2) characterize linear actuators; (3) evaluate volume control; (4) evaluate pressure generated by mouth-pipetting and SAID, and (5) evaluate the feasibility of sample delivery into female live-bearing fish. The SAID can be deployed within the research community through open sharing and thus facilitate standardization and participation in the use of cryopreserved genetic resources.

## 2. Materials and Methods

### 2.1. Design and Fabrication

Prototypes of SAID were assembled with mechanical and electrical components, with control of fluid transfer and volume based on an air displacement mechanism produced by the movement of a piston [32]. Standard glass capillary tubes were chosen as disposable components because of their wide availability. There were several design considerations for the SAID: (1) it should accommodate commercially available capillary tubes (Drummond Scientific Company, Broomall, PA) with calibrated 10 µL volumes (outer diameter = 1.041 mm); (2) it should be able to transfer 1–5 µL of fluid with a variation of <10% of the target volume; (3) the maximum pressure generated during insemination should be equal to or lower than those produced by mouth-pipetting operators; (4) it should be operable with one hand (with the other hand used to manipulate the fish) and be connected to a foot pedal (switch) to control fluid transfer, and (5) the overall material cost of the device should be <USD 100.

Two types of consumer-level three-dimensional (3-D) printers were used to fabricate components: a stereolithography (SLA) 3-D printer (Photon S, Anycubic, Shenzhen, China) that printed photopolymerizable resins (Anycubic), and a fused deposition modeling (FDM) printer (SV01, Sovol Technology Co., Hong Kong, China) configured for polylactic acid (PLA) filament (ZYLtech Engineering, Houston, TX, USA). Designs were created by use of computer-aided design (CAD) software (Inventor 2021, Autodesk, San Rafael, CA, USA). The 3-D models were converted to stereolithography (STL) files, which were imported to slicing software (Ultimaker Cura 4.9.1, Ultimaker B.V., Zaltbommel, the Netherlands) for FDM printing (Table S1) and Photon Workshop (Anycubic) for SLA printing (Table S2). Printing settings were defined in the slicer software and converted to G-Code format for printing. After SLA printing, components were removed from the printing platform and rinsed thoroughly by use of an ultrasonic bath (HB-4868, H & B Luxuries, accessed from Amazon.com) in 99.5% isopropyl alcohol (Sigma-Aldrich, St. Louis, MO, USA), followed by post-printing curing with a UV lamp (DR-301C, MelodySusie, Union City, CA, USA) for 5 min. All components were printable with the SLA printer, but the FDM printer was used to reduce printing time for larger components (described below).

### 2.2. Characterization of Linear Actuator

A micro linear actuator (GS-1502, Goteck, Guangdong, China) was used to control air displacement for flow transfer. This actuator converted the rotational motion of a servo motor to linear travel of a servo horn (Figure S1). The servo motor was controlled with an Arduino Nano (Arduino, Somerville, MA, USA) microcontroller with a program (written with Arduino IDE 1.8.15) by applying command pulses via pulse width modulation (PWM), where input pulses between 900 μs and 2100 μs corresponded to target angles of the servo motor. A 900 μs pulse moved the internal servo motor to the minimum position (0°), and a 2100 μs pulse moved it to the maximum position (180°) [33]. The programmed codes (File S1) converted the command pulses to percentages of the maximum stroke of linear displacement of the actuator (referred to as “programmed position” in percentage), where 0% was fully retracted (0 mm), and 100% was fully extended (7 mm) [34]. The SAID loaded samples when the actuator retracted, held samples when the actuator was static, and ejected samples when the actuator extended. To prevent motor damage, the actuator operated between 5% (0.35 mm) and 95% (6.7 mm) of this range. The control circuitry (Figure S1) for the SAID included two pushbuttons, two 10 kΩ resistors, the linear actuator, and a rechargeable battery.

The output volume and pressure of the SAID were controlled by air displacement driven by the piston. Therefore, the relationship between the programmed position and the actual position of the servo horn was characterized. A measurement adapter (Figure S1) was created to provide a flat edge over the servo horn to allow measurement of the positions with a caliper. The servo horn was programmed to positions of 10% (“initial position”) to 90% in 10% increments (and 10 replicates for each position). The actuator displacement of the servo horn was expressed as: (position of each 10% increment) − (the initial position).

### 2.3. Evaluation of Fluid Volume Delivery

The relationship between the programmed position of the linear actuator and the volume transferred by the SAID was evaluated. To test the loading accuracy of a known volume of sample, the end of a 10 μL capillary was placed in 1 mL of water stained (to facilitate visual observation) with blue food dye (Wilton Industries, Woodridge, IL, USA) in a Petri dish. The initial programmed position was set at 90% and tested for retraction distance in increments of 10% as described above. After the testing of each retraction distance, the actuator was programmed to return to the original position (90%), and a new capillary tube was used (with 10 replicates). The volume drawn into the capillary tube was assessed with an image analysis method with software Fiji (https://imagej.net/software/fiji/, accessed on 4 May 2022) [35]. Pre-manufactured 10 µL marks on the capillary tubes were used for image calibration. The fluid volume drawn by SAID was calculated as: 10 µL × (length of fluid column/length of 10 µL mark).

### 2.4. Evaluation of Pressure

Mouth-pipetting is the most commonly used method for artificial insemination of small-bodied live-bearing fishes, but there are no reports characterizing the pressure range produced with this method. The breath pressure generated by eight operators practicing mouth-pipetting was evaluated with a digital manometer (HHC280, Omega Engineering, Newark, CT, USA). The mouth-pipettor (Drummond Scientific Company) was connected to the manometer and capillary tubes with a “T”-junction fitting (Figure 1). Volumes were labeled based on the distance from the tip of capillary tubes to the 10 µL mark. Eight operators (one with experience in mouth-pipetting and seven without experience) conducted the testing by loading water samples (colored with blue food dye) with target volumes of 3, 5, and 10 µL into capillary tubes and ejecting them into a Petri dish. The operators were instructed to dispense samples in two scenarios. For the first scenario, operators imagined samples were inseminated into female fishes by ejecting as gently as possible. For the other scenario, operators ejected samples as fast as possible (with stronger exhalation). Ten replicates were made with each sample volume and scenario. Peak pressures displayed by the manometer were recorded.

A differential pressure sensor (AMS 5812-0004-D, Analog Microelectronics, Mainz, Germany) was used to characterize dynamic pressure changes. This sensor could be sampled at a maximum rate of 1000 samples/s in the range of 0 to 0.4 psi with a maximum reported error of 2%. The digital manometer (Omega Engineering) for estimation of mouth-pipetting described above had a sampling rate of 1 sample/s and thus was only used for peak pressure estimation and not for characterization of dynamic pressures. Prototypes of SAID were assembled and connected to the pressure sensor with “T”-junction fittings (Figure S2). The pressure sensor was integrated with the Arduino control board (Figure S3) for control (File S1) and recording of pressures (File S2). Real-time pressure measurements were recorded at 50 ms intervals during loading, holding, and ejecting of water samples. For peak pressure assessment, the initial programmed position of the actuator was set at 10% and displaced by moving to programmed positions from 10 to 80% in 10% increments, repeated 10 times. To characterize dynamic pressure, the SAID was programmed to load and eject 5 μL (60% programmed position) of water with 10 replicates.

### 2.5. Evaluation of Sample Delivery into Female Live-Bearing Fish

The feasibility of insemination for live-bearing fish with SAID prototypes was evaluated by dispensing isotonic Hanks’ balanced salt solution (HBSS) at 300 mOsmol/kg (commonly used to suspend sperm samples for aquatic species) [36] into the reproductive tracts of redtail splitfin (*Xenotoca eiseni*). Protocols for the use of animals in this study were reviewed and approved by the Louisiana State University Institutional Animal Care and Use Committee. The *X. eiseni* used in this study were cultured at the Aquatic Germplasm and Genetic Resources Center (AGGRC) of the Louisiana State University Agricultural Center within an 800 L recirculating water system with water quality monitored weekly [22].

Female fish were anesthetized with 0.02% tricaine methanesulfonate (MS-222, Western Chemical, Inc. WA, USA). The standard lengths were 40 ± 3 mm (mean ± SD), and body weights were 2.0 ± 0.5 g. The anesthetized females were placed on their backs on a centrally hollowed sponge on a dissection scope stage. A capillary tube containing HBSS was inserted about 2 mm into the reproductive tract for fluid ejection (Figure 1). Samples with volumes of 1, 3, and 5 μL (based on the relationship between volume and programmed position of the actuator) were dispensed into 10 females for each volume (with an additional 10 females without insemination as a control group). Females were inspected visually for injuries after insemination and returned to separate tanks for recovery. Females were cultured and observed for 30 d after insemination.

## 3. Results

### 3.1. Design and Fabrication

**Figure 2:**
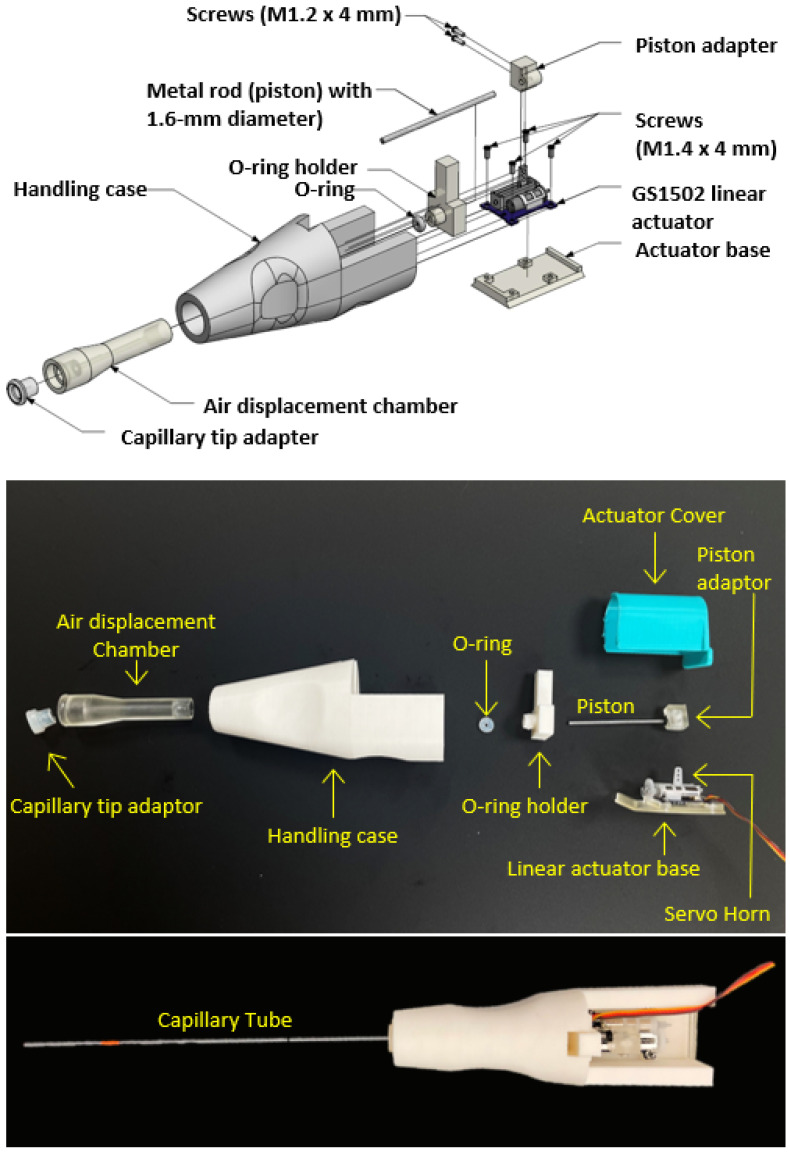
Assembly of the prototype standardized artificial insemination device (SAID). A 3-D rendering (**upper**), fabricated components (**middle**), and an assembled prototype (**lower**) of SAID.

The SAID assembly (Figure 2) was composed of five 3-D printed components and several commercially available components (sources are provided in Table 1), including a linear actuator, six screws, a metal rod, an O-ring, and a capillary tip adapter. A 3-D printed piston adapter connected the servo horn to the metal rod (1.6 mm in diameter, 40 mm in length). The metal rod served as a piston (resembling a syringe plunger,) moving back and forth by the actuator, and creating pressure changes due to air displacement inside the 3-D printed chamber. A rubber O-ring created a seal between the metal rod and air displacement chamber, and an O-ring holder stabilized the O-ring in the chamber. A servo base positioned the actuator inside a handling case with ergonomic features that secured the internal components and facilitated user handling. An actuator cover was designed to protect the electrical components from damage. A capillary tip adapter provided sealing between the air displacement chamber and capillary tubes. Target volumes were selected with a rotary button on the user interface, and fluid movement was initiated with a foot pedal. The total material cost of a single SIAD prototype was about USD 73 in 2020–2021 (Table 1).

### 3.2. Characterization of Linear Actuator and Volume Control

**Figure 3:**
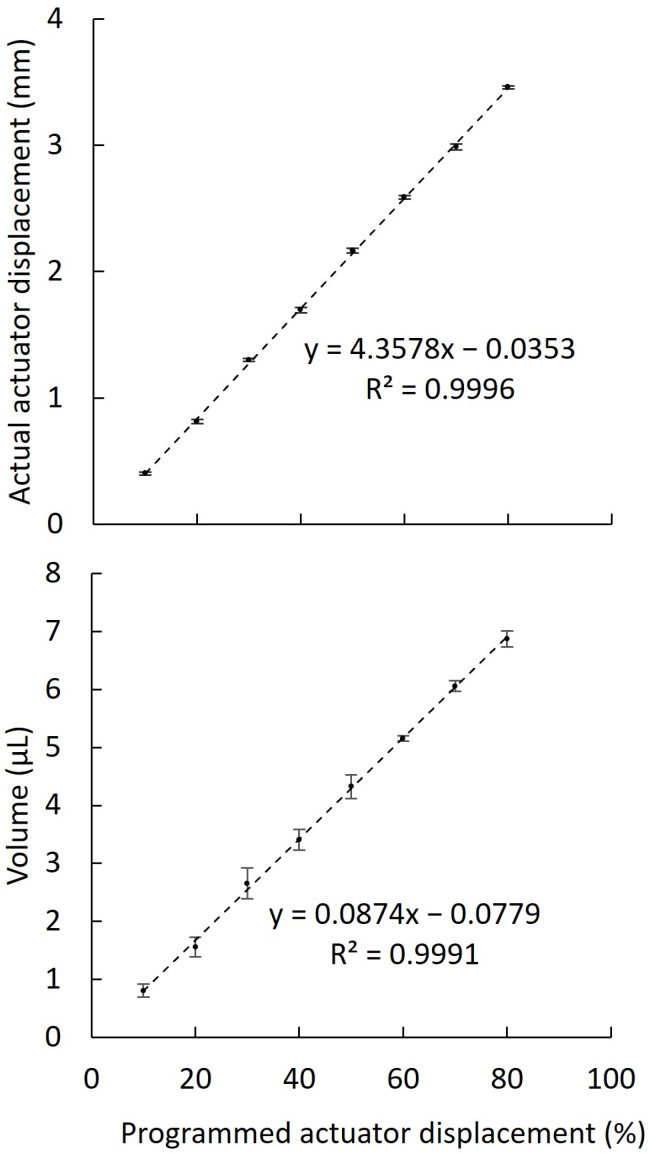
Characterization of a low-cost linear actuator for volume control with the standardized artificial insemination device (SAID). There were linear relationships between the programmed and actual positions of the linear actuator (**upper panel**) and between the programmed position and volume of sample transfer (**lower panel**).

There was a linear (*r^2^* = 0.9996) relationship (Figure 3) between the programmed actuator displacement and the actual actuator displacement, with about 4 mm of actual displacement accounting for each 10% portion of programmed displacement. The standard deviation of each actual displacement was 0.04–0.08 mm. There was a linear (*r*^2^ = 0.9991) relationship (Figure 3) between the programmed actuator displacement and sample volume expelled by the SAID. A prediction equation was defined as: Volume (μL) = (0.0874 × programmed actuator displacement) − 0.0779. This equation was programmed in the user interface to allow the selection of the target volume of sample transfer.

### 3.3. Evaluation of Pressure

**Figure 4:**
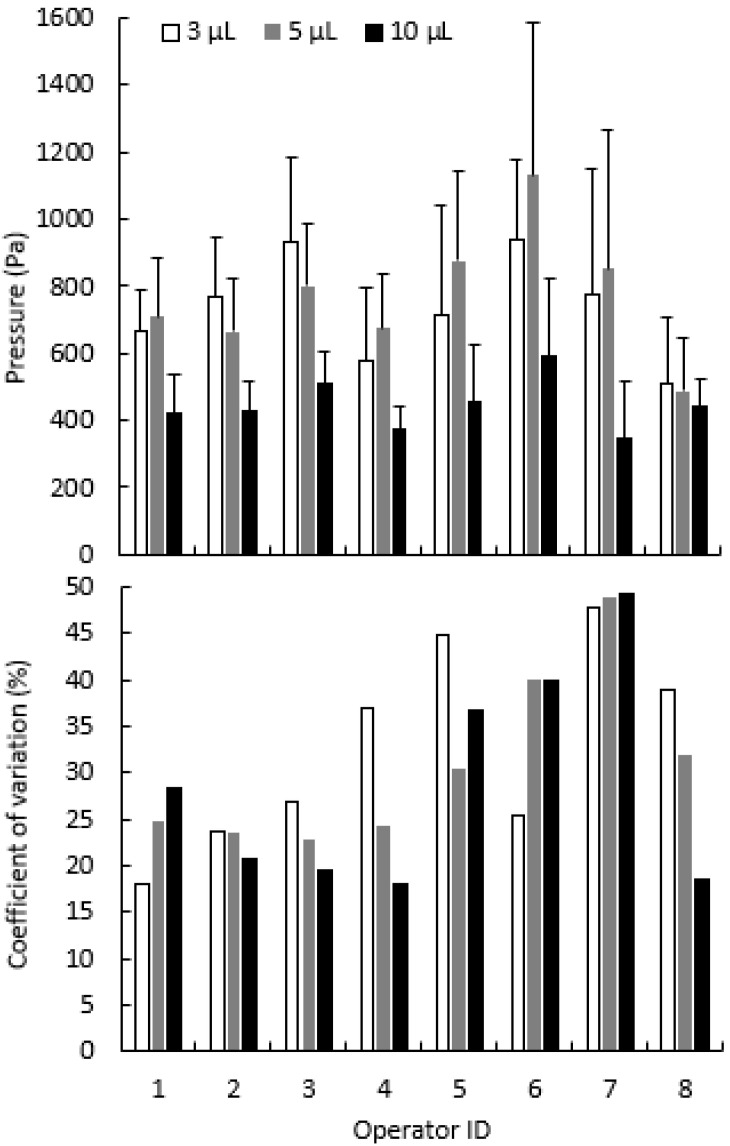
Peak pressure (**upper panel**) and variability (**lower panel**) generated during mouth-pipetting among eight operators when asked to simulate injection of samples into small-bodied live-bearing fishes. Bars represent standard deviation.

When asked to eject samples with gentle mouth-pipetting, eight individuals generated different dispensing pressures (Figure 4) for 3 µL (*p* = 0.0009), 5 µL (*p* = 0.0002), and 10 µL (*p* = 0.0071). The breath pressure generated among all users ranged from 511 to 938 Pa when ejecting 3 μL, 488 to 1131 Pa for 5 μL, and 344 to 591 Pa for 10 μL. The pressures generated by individuals varied among the 10 replicates, with coefficients of variation from 18% to 48% for 3 μL, 20% to 49% for 5 µL, and 18% to 49% for 10 µL. When asked to eject samples as fast as possible (Figure S4), the pressure generated ranged from 555 to 5319 Pa, with the coefficients of variation ranging from 25% to 112%. The standard deviation ranged from 67 to 453 Pa for gentle pipetting and ranged from 139 to 2178 Pa for fast pipetting.

**Figure 5:**
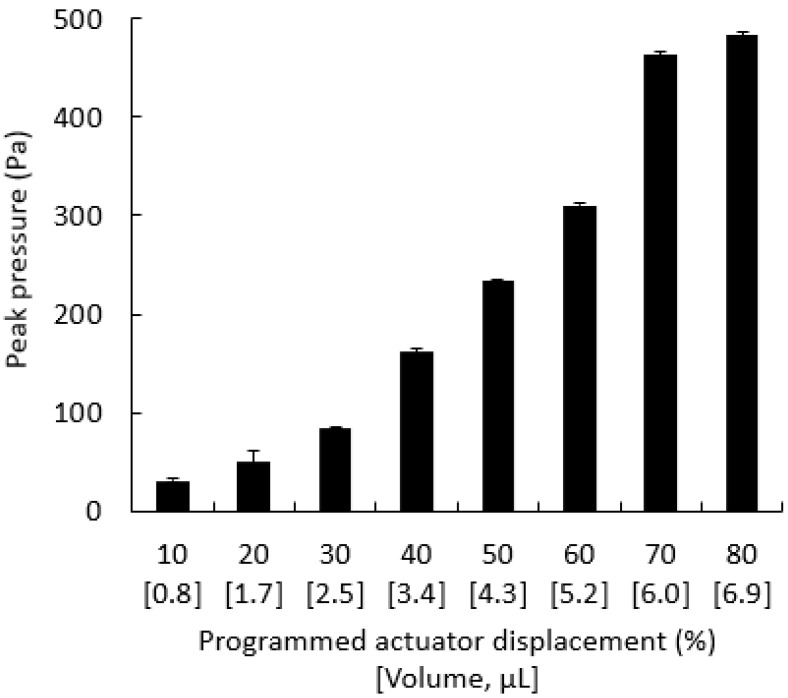
Peak pressure generated by prototypes of the standardized artificial insemination device (SAID) during sample delivery of various volumes. Programmed actuator displacement of 10–80% controlled sample volumes of 0.8–6.9 µL.

The peak pressures generated (Figure 5) by SAID prototypes ranged from 31 Pa to 483 Pa for programmed actuator displacement of 10–80%. The coefficient of variation of peak pressures was <2% for displacement of 30% to 80% (volumes of 2.5–6.9 μL), lower than those generated by mouth-pipetting in the same volume range. The coefficient of variation for 10% to 20% programmed displacement (0.8–1.7 µL) ranged from 8% to 22%. The standard deviation of pressures generated by SAID was <11 Pa for all volumes.

**Figure 6:**
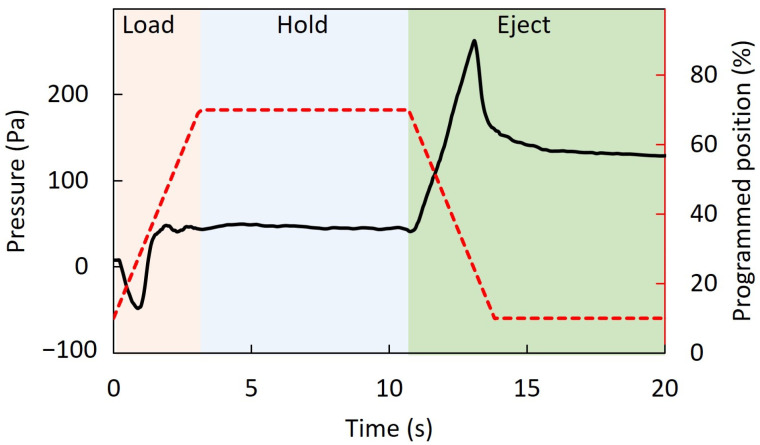
Dynamic pressures (left axis) generated by a prototype standardized artificial insemination device (SAID) during loading, holding, and ejecting of 5 μL water samples. Programmed actuator position is shown on the right axis.

The dynamic pressure (Figure 6) during liquid transfer showed momentary negative pressures (−48 Pa) prior to fluid filling during retraction of the linear actuator, followed by stable positive pressures when the actuator was programmed to be static. When the actuator pushed the piston to the original position, the positive pressures momentarily increased about 100 Pa at peaks during initial ejecting, and then remained stable. A small volume (<0.5 µL) of fluid was observed remaining in the tips of capillary tubes after completion of each ejection.

### 3.4. Evaluation of Sample Delivery into Female Live-Bearing Fish

The SAID was able to reliably eject fluid volumes of 1, 3, and 5 µL into the female reproductive tracts of redtail splitfin. No visible injuries were observed around injection regions. After 30 days, no mortality or deformities were observed within the three testing groups or the control group.

## 4. Discussion

Mouth-pipetting has served as the major method for artificial insemination for small-bodied live-bearing fishes for the past 70 years. This method has been preferred over the use of a micropipettor because both hands are free for the positioning of fish and capillary tubes while controlling the fluid flow with breath pressure. In addition, micropipettors often cause relatively higher mortality because of the challenges in performing delicate movements with the wrist and in sensing the interaction between the reproductive opening and pipette tip (personal communication with XGSC). However, mouth-pipetting has been eliminated from daily practices in most laboratories because of risks of contamination and health hazards [37]. Mouth-pipetting for insemination has specific problems, such as the requirement of a high skill level based on expert training and considerable experience, risk of injury to female fishes, and unstandardized control of air pressure and sample volume. These problems impede the broad utilization of genetic resources and inhibit the direct comparison of research outcomes. In the present study, SAID prototypes were developed to address issues with mouth-pipetting while providing the capabilities of hands-free pressure control, standardization, and customization.

### 4.1. Design and Fabrication

Prototypes were designed as open hardware that can be accessed, fabricated, and assembled by users at low cost with entry-level skills in 3-D printing and electronics. The flow control mechanism resembled those used in air displacement pipettes [31] and syringe pumps [38]. This mechanism was controlled by the positioning of a metal rod as a piston within an airtight displacement chamber. Consumer-level SLA 3-D printing was chosen to fabricate the displacement chamber because it can form airtight structures more reliably than fabrication with FDM printers [39]. In addition, SLA-printed components provide better surface smoothness within the chamber that facilitates piston movement [40]. While components fabricated with some resins can be prone to abrasion and material degradation [41], no apparent wear was observed in these studies (<200 repetitions of use). Methods to optimize strength and surface characteristics over prolonged use could be evaluated in future studies, including adjustment of the print settings and post-processing steps [42,43]. The addition of a foot pedal freed both hands for use in fish handling and insemination. The foot pedal did not adjust pressure and volume but served as a switch to engage load or inject commands to the device.

Based on the prototypes developed in the present work, design and fabrication modifications could be made to improve the functionality of SAID prototypes. In the present study, the capillary tip adaptor, which enhanced the sealing of the air displacement chamber, was a piece of elastic silicone that was commercially available with the purchase of a pack of capillary tubes (Drummond Scientific). In future studies, elastic components (e.g., the capillary tip adaptor and O-rings) could be fabricated by 3-D printing with flexible resins [44] or filaments [24] to reduce reliance on commercial components. The capillary tube adaptor could be further customized to fit other liquid transfer items, such as pipette tips or blunt syringe needles. In addition, printed circuit boards (PCB) can be designed in the future to make the assembly more compact [27].

### 4.2. Linear Actuator Characterization

Various pumping systems can be used for fluid control in scientific hardware, such as peristaltic pumps [45], diaphragm pumps [46], and syringe pumps [38]. A piston pump controlled by a linear actuator was used for the SAID prototypes because of their low cost (USD 7), amenability to open-hardware application, and a miniaturized size profile enabling compact assembly. Linear actuators are commonly used in the development of mechanical and robotic systems [47,48]. A highly linear relationship (*r*^2^ > 0.99) was observed between the programmed and actual displacement of the actuator, enabling positional control of the piston to accurately manipulate fluid transfer. Minimal variation in positional control (i.e., standard deviation <0.1 mm) was a prerequisite for accurate volume control. In future studies, features such as the speed of actuator displacement could be evaluated to potentially control fluid flow rate, if that parameter were deemed important to specific biological research applications.

### 4.3. Volume Control

The volume of fluid extracted into capillary tubes had a strong (*r*^2^ > 0.99) linear relationship with programmed actuator displacement. With this relationship, the target volume of fluid transfer can be programmed (e.g., in 1 µL increments) through the user interface prior to inseminator operation. This relationship can be affected by the dimensions of capillary tubes, pistons, and air displacement chambers. In addition, several factors can affect the captive air volume within the air displacement chamber, including barometric pressure, liquid density, liquid viscosity, external humidity, and temperature [31]. As such, the establishment of calibration curves with the methods developed herein would be advisable when such devices are used in different operational conditions. Although the volume capacity was evaluated based on the insemination of small-bodied live-bearing fishes [22], SAID prototypes could be modified with larger actuators and components for use with larger animals such as reptiles, amphibians, birds, and mammals.

### 4.4. Pressure Generation

Unstandardized pressure control using traditional mouth-pipetting methods of artificial insemination presents several issues. Relatively high pressures can damage the female reproductive tract or ovaries, whereas relatively low pressures can yield inadequate delivery of sperm. Pressures generated with mouth-pipetting by eight operators indicated the potential for considerable variation within and among individuals. Because it was challenging to cease exhalation by mouth at the exact moment when fluid was completely ejected from the capillary, extended application of positive pressure was observed. Sample dispensing with SAID prototypes was designed to address these issues. Pressures generated by SAID were smaller than those produced by mouth-pipetting, and it eliminated the extended pressure after completion of sample transfer, reducing the risk of injury to valuable female fishes. In addition, the small pressure variations of the SAID can facilitate the direct comparison of fertilization outcomes among laboratories, greatly enhancing research reproducibility [49]. Such improvements in reproducibility are of high priority to research agencies such as the National Institutes of Health, which provide significant funding for biomedical research [49].

Results of dynamic pressure measurements during loading and dispensing revealed that momentary vacuum pressures were generated during initial loading, followed by stable pressures during the hold phase of operation, and momentarily increased during initial ejecting. These momentary changes in pressure can be attributed to the combination of capillary resistance to fluid flow and the dynamic pressures changes during the initial stages of volume expansion during loading and contraction, similar to other fluid delivery systems [50]. The small residual volumes of fluid observed in the capillary tube after ejection were also likely attributed to capillary action and were not found to affect the volume of sample transferred. Dynamic pressure signals could be monitored in future iterations of the device as an indication of fluid displacement. As such, pressure sensors could be integrated into the SAID assembly in future designs to monitor, record, and standardize the sample transfer process. In addition, manipulation of peak pressure by altering the actuator speed could be further investigated for biological applications, where the flow rate of insemination procedures is deemed important. The effects of sample viscosity on pressure and flow rate could also be further investigated.

### 4.5. Sample Delivery into Females

Mortality is common among inseminated females even when mouth-pipetting is performed by well-trained technicians. Possible factors that can cause mortality include handling stress, anesthesia, catheters (capillary tubes or pipette tips), insemination depth, sample volume, and pressure. The present study directly addresses sample volume and pressure and can indirectly reduce handling stress. Prototypes were able to create pressures sufficient to deliver 1–5 μL of fluid into the reproductive tracts of redtail splitfin females yet remained low enough to avoid physical injuries. Glass capillary tubes were chosen as disposable components for sample transfer because they are standardized among manufacturers and can be widely accessible. Additionally, the tips of glass capillary tubes can be polished by heating to produce rounded edges that could further reduce the risk of injury. In the present study, the 2 mm insertion depth was estimated by operators. In future designs, stoppers (or markers) could be added to the outer diameter of capillary tips to control depth of insertion into the reproductive tract. Overall, inseminator devices such as these would enable future study of the effects of insemination pressure, volume, and flow rate on fertilization rate for live-bearing fishes. Such studies were beyond the scope of the present work, which was intended to develop prototypes and evaluate feasibility.

## 5. Conclusions

A standardized artificial inseminator device (SAID) was developed, prototyped, and evaluated for use with small-bodied live-bearing fishes. The device was designed as open hardware at low cost (<USD 80) using commercially available and 3-D printed components to enable broad community access. A user interface was developed to allow feature selection, and a foot pedal was connected to free the hands for operation. A linear actuator was used to accurately control the positioning of a piston for fluid transfer, with a standard deviation of <0.1 mm for 4 mm intervals. The volume of sample transfer was precisely controlled by programming the linear actuator with a linear relationship (*r*^2^ > 0.99) between the piston position and volume. The pressure profiles generated by the inseminator prototypes were superior to those generated by mouth-pipetting, yielding lower overall pressures (31–483 Pa) and variation (standard deviation <11 Pa). Delivery of 1–5 μL of fluid into female reproductive tracts was accomplished with the SAID and resulted in no physical injuries or mortality. Problems of existing mouth-pipetting procedures can be addressed with the inseminator developed in this study, which can enable standardized insemination with minimal training, and improve participation of research communities and the use of genetic resources. Further studies can be made to improve functionality and evaluate the feasibility of the application of this device in other animals or applications.

## Figures and Tables

**Table 1:** Bill of materials including source and cost of components used for prototypes of the Standardized Artificial Insemination Device (SAID).

Item	Printing Material	Vendor	Price (USD)	Quantity	Cost (USD)
Air displacement chamber	Resin	Anycubic ^a^	40.00/L	2.304 mL	0.09
Piston adapter	Resin	Anycubic ^a^	40.00/L	0.273 mL	0.01
Actuator base	Resin	Anycubic ^a^	40.00/L	0.853 mL	0.03
Handling case	PLA	Hatchbox ^b^	24.99/kg	17.6 g	0.44
O-ring holder	PLA	Hatchbox ^b^	24.99/kg	0.9 g	0.02
O-ring		Amazon ^c^	0.27/ct	1 ct	0.27
Metal rod (piston)		Amazon ^c^	0.37/ct	1 ct	0.37
Linear actuator (GS-1502)		Amazon ^c^	7.00/ct	1 ct	7.00
Arduino Nano		Arduino ^e^	15.99/3ct	1 ct	6.00
Monochrome OLED (1.3”)		Adafruit ^f^	19.95/ct	1 ct	19.95
Rechargeable Battery (Anker PowerCore, 5 V)		Amazon ^c^	16.19/ct	1 ct	16.19
Foot Pedal (MLCS 9080)		Amazon ^c^	22.95/ct	1 ct	USD 22.95
Total					USD 73.32

Link for purchase: ^a^ https://www.anycubic.com; ^b^ https://www.hatchbox3d.com; ^c^ https://www.amazon.com; ^e^ https://www.arduino.cc/; ^f^ https://www.adafruit.com/product/938. All accessed on 4 April 2022.

## Data Availability

Design files will be available on AGGRC webpages upon completion of prototype testing.

## Supplementary Materials

The following supporting information can be downloaded at: https://www.mdpi.com/article/10.3390/ani12080961/s1, Figure S1: The linear actuator (upper left) used in prototypes of the standardized artificial insemination device (SAID) and a 3-D rendering of an adaptor (upper right) to assist measurement of the position the servo horn. A circuit (bottom) was used to control the linear actuator with rotary buttons; Figure S2: Configuration used for pressure measurement of a prototype standardized artificial insemination device (SAID); Figure S3: Circuits for control and user interface of a prototype standardized artificial insemination device (SAID) based on use of the Arduino Nano microcontroller; Figure S4: Peak pressure (upper panel) variability (lower panel) generated during mouth-pipetting among eight operators when asked to inject samples as quickly as possible. Bars represent standard deviation; Table S1: Specifications for FDM printing of the handling case, interlocking clip, servo case, and user interface case; Table S2: Specifications for resin printing of the cylindrical chamber, piston adaptor, and linear actuator base; File S1: Arduino programming codes for SAID operation; File S2: Arduino programming codes for the AMS pressure sensor.
