# Supplementary material for: An Open-Hardware Insemination Device for Small-Bodied Live-Bearing Fishes to Support Development and Use of Germplasm Repositories
Source: Animals (Basel). 2022 Apr 8;12(8):961. doi: 10.3390/ani12080961 (PMC9032428; doi:10.3390/ani12080961)
Supplement: Supplementary file 1 [file animals-12-00961-s001.zip › Supplementary-Tables & Figures.pdf]

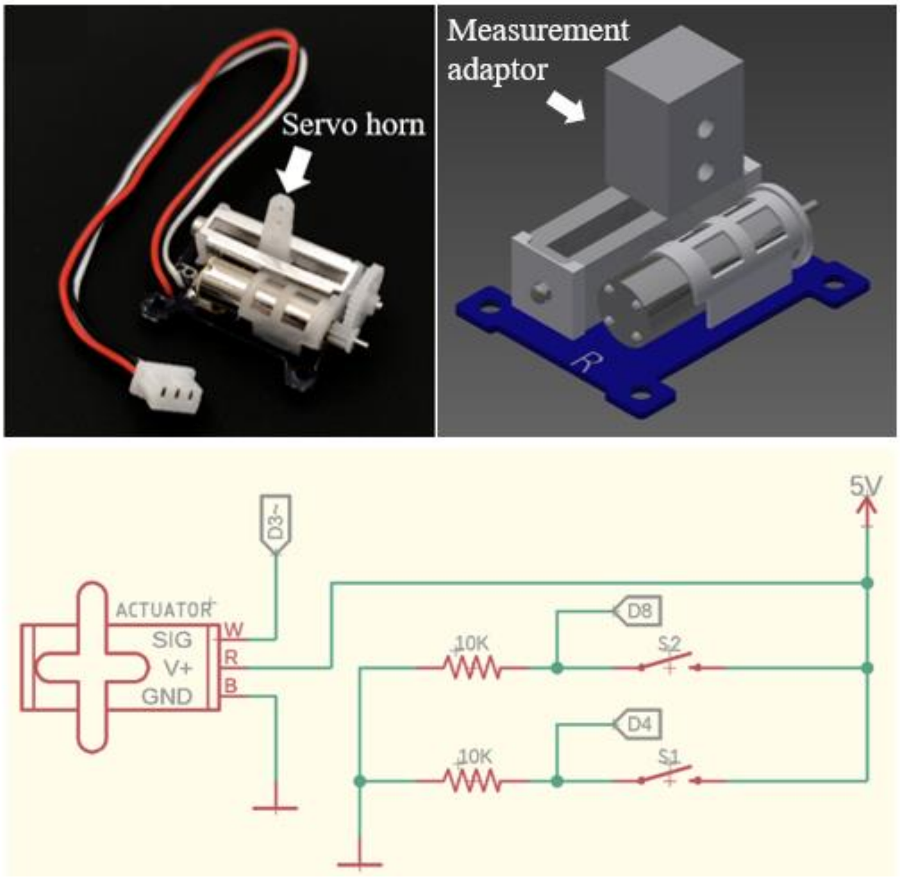

Supplemental Figure S1. The linear actuator (upper left) used in prototypes of the standardized artificial insemination device (SAID) and a 3-D rendering of an adaptor (upper right) to assist measurement of the position the servo horn. A circuit (bottom) was used to control the linear actuator with rotary buttons.

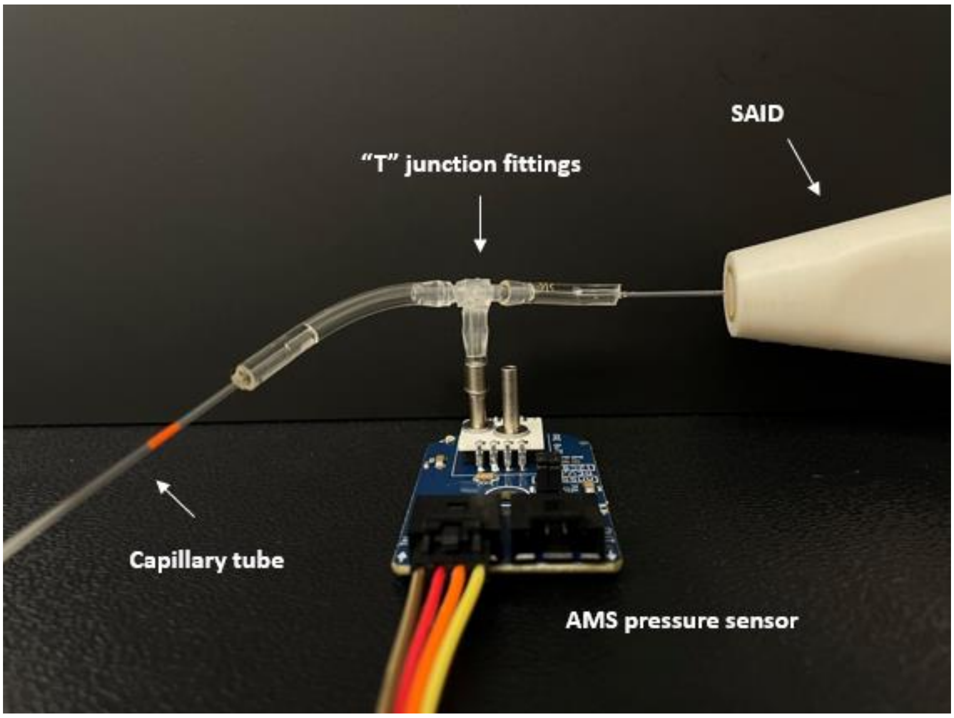

Supplemental Figure S2. Configuration of pressure measurement of a prototype standardized artificial insemination device (SAID).

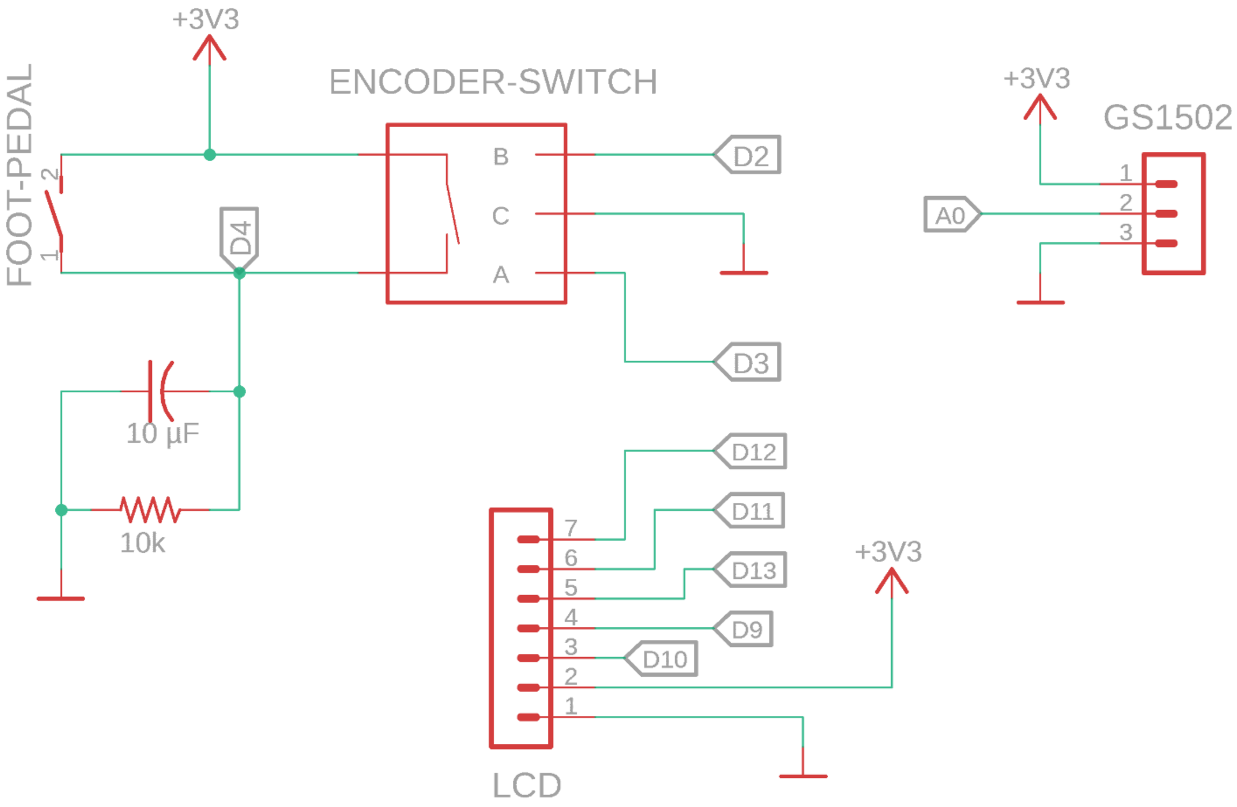

Supplemental Figure S3. Circuits for control and user interface of a prototype standardized artificial insemination device (SAID) based on use of the Arduino Nano microcontroller.

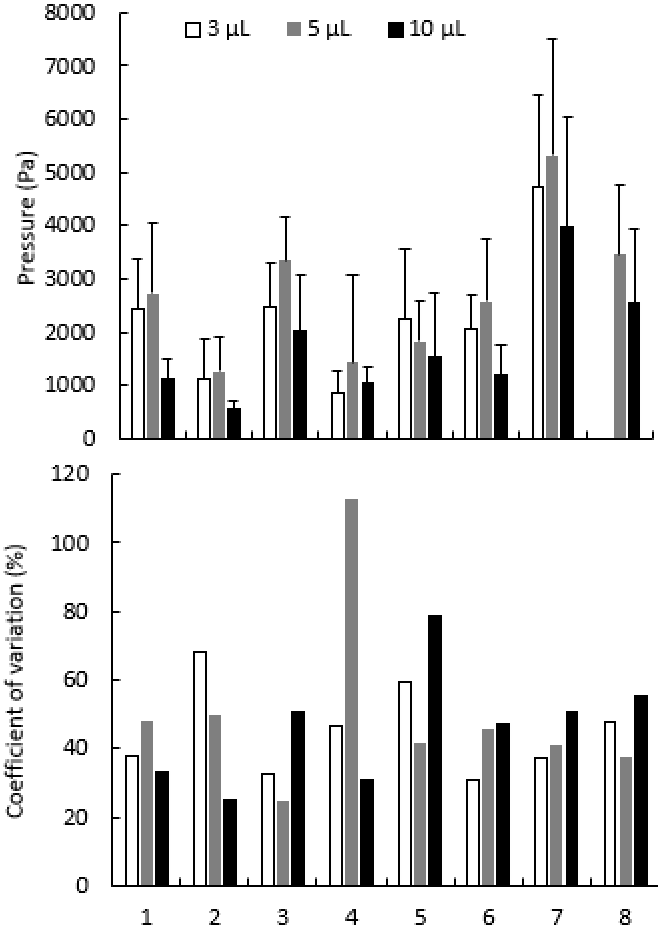

Supplemental Figure S4. Peak pressure (upper panel) variability (lower panel) generated during mouth-pipetting among eight operators when asked to inject samples as quickly as possible. Bars represent standard deviation.

Supplemental Table S1. Specifications for FDM printing of the handling case, interlocking clip, servo case, and user interface case.

Parameters	Specifications
General settings:	
Hotend temperature	200°C
Print speed	50 mm/s
Nozzle type	Copper
Nozzle diameter	0.4 mm
Line width	0.4 mm
Nominal layer height	0.12 mm
Retraction distance	5 mm
Retraction speed	45 mm/s
Build plate temperature	50 °C
Fan speed	100%
Perimeter layer number	2
Top layer number	2
Bottom layer number	2
First layer settings:	
Build plate adhesion	Raft
Raft air gap	0.2 mm
Raft top layers	3
Raft print speed	25 mm/s
Raft fan speed	0%
Initial layer speed	20 mm/s
Initial layer height	0.12 mm
Initial line width	0.45 mm

Supplemental Table S2. Specifications for resin printing of the cylindrical chamber, piston adaptor, and linear actuator base.

Parameter	Specifications
Print settings:	
Layer height	0.05 mm
Normal exposure time	15 s
Off time	6 s
Bottom layers	8
Bottom exposure time	40 s
Post-processing steps:	
Ultrasonic bath	2 min
Solvent	99.5% Isopropyl alcohol
UV curing time[a]	5 minutes
